# Supplementary material for: Hydrazine High-Performance Oxidation and Sensing Using a Copper Oxide Nanosheet Electrocatalyst Prepared via a Foam-Surfactant Dual Template
Source: Nanomaterials (Basel). 2022 Dec 26;13(1):129. doi: 10.3390/nano13010129 (PMC9823773; doi:10.3390/nano13010129)
Supplement: Supplementary file 1 [file nanomaterials-13-00129-s001.zip › nanomaterials-2066666-supplementary.pdf]

nanomaterials

Supplementary Materials

Hydrazine High-Performance Oxidation and Sensing Using Copper Oxide Nanosheet Electrocatalyst Prepared via Foam-Surfactant Dual Template

Etab M. Almutairi, Mohamed A. Ghanem *, Abdulrahman Al-Warthan, Mufsir Kuniyil and Syed F. Adil

Chemistry Department, College of Science, King Saud University, Riyadh 11451, Saudi Arabia
* Correspondence: mghanem@ksu.edu.sa; Tel.: +966-114670405; Fax: +966-14675992

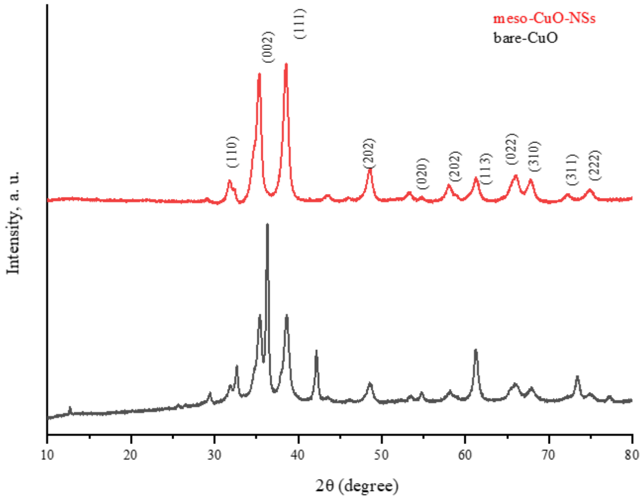

Figure S1. XRD of CuO-NSs and *bare*-CuO.

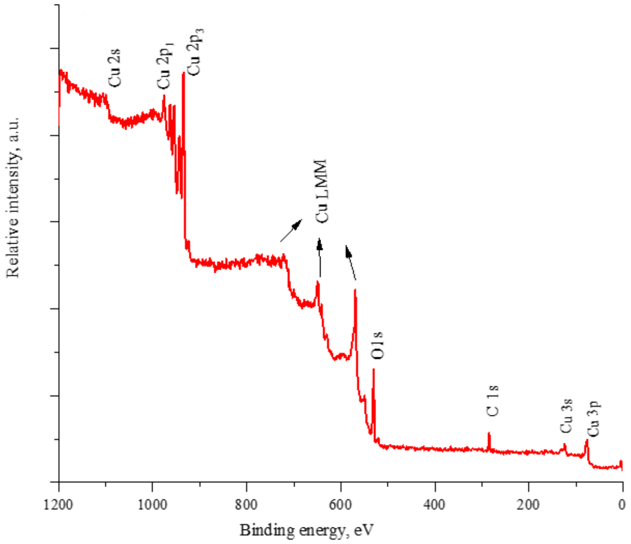

Figure S2. The XPS survey spectrum of as-prepared CuO-NS.

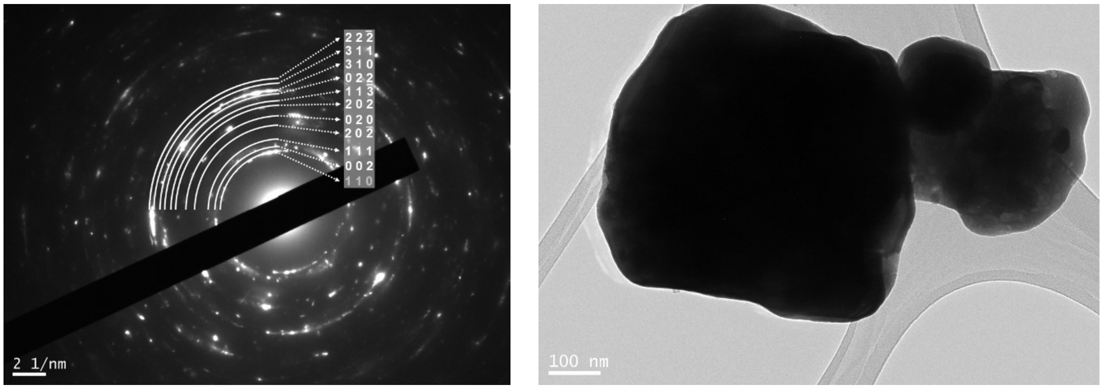

(**a**) (**b**)

Figure S3. (**a**) the SAED pattern of the CuO nanosheets, and (**b**) TEM image of the *bare*-CuO catalyst deposited in the absence of the surfactant.

Table S1. Results for detection of hydrazine from different water sources injected with known concentration of hydrazine.

Sample	Determined Real Sample (μM)	Hydrazine Spiked (μM)	Hydrazine Recovered (mM)	Mean of Hydrazine Recovered (mM)	RSD [a] (%)	Recovery [b] (%)
Tap water	0.00	0	0.00			
		1	1.13, 1.07, 1.05	1.083 ± 0.0339	3.1378	108.3
		2	2.21, 2.03, 2.18	2.13 ± 0.0787	3.696717	106.5
		3	3.23, 3.24, 3.233	3.234 ± 0.0053	0.164167	107.66
		4	4.0, 4.076, 4.0	4.024 ± 0.0367	0.913756	100.65
		5	4.74, 4.97, 4.876	4.864 ± 0.0935	1.923271	97.285
		6	6.0, 5.71, 5.76	5.8525 ± 0.125	2.15714	97.08
Bottled water	0	0				
		1	1.15, 1.0, 1.1	1.063 ± 0.0531	4.99	106.33
		2	2.15, 2.13, 2.08	2.12 ± 0.029	1.38	106
		3	3.0, 3.0, 3.13	3.043 ± 0.061	2.0139	101.44
		4	4.0, 4.0, 4.06	4.02 ± 0.028	0.704	100.5
		5	5.0, 5.0, 4.876	4.958 ± 0.058	1.179	99.173
		6	5.98, 6.0, 6.0	5.995 ± 0.007	1.25	99.911

[a] RSD: relative standard deviation for three independent measurements; [b] Recovery = (C predicted)/(C spiked) × 100.
